# Our Contemporaries

**Published:** 1915-11

**Authors:** 


### OUR CONTEMPORARIES.

---

The Pacific Medical Journal fills small vacant spaces with quotations from the Bible. The Bible is the most extensively printed, if not the most widely read book in the world. If it were not so generally regarded as a religious work, many persons would read it who do not. We were surprised, some time ago, to hear a surgeon who is not especially pious, say that he sat up till 1 o'clock reading the old testament because of its fascinating interest.

---

The Cincinnati Medical News declares that many persons recorded as dying from alcoholism, really die from too liberal doses of chloral, given therapeutically.

---

The Critic and Guide prints the parable of the young man with a pornographic mind who had an idea. Briefly, finding that the cost of indecent pictures and literature was becoming a serious drain on his purse, he organized himself into a society for the prevention of vice and thus obtained a fine collection, free. It occurs to us that such an article should have been delayed a few months unless the editor is willing to forego the rule of De mortuis nil nisi bonum and to make definite charges. We would scarcely go so far as to agree that vice hunting is merely one variety of sexual perversion, although it sometimes seems as if the purity specialists get too much interested in sex.

---

Bulletins et Memoirs de la Societe de Medecine de Paris, June 23, devotes over 20 pages to a dissertation on the Polychesia of the German Race, by Dr. Berillon. Some historic notes of the interest shown by Martin Luther and a German princess in defaecation are given, and the claim is made that the faeces of Germans are double in amount those of the French and much more offensive. It is gravely stated that 500 German cavalrymen, in 3 weeks, deposited 30,000 kilos of dejecta in the various rooms of a paper factory in Chenevieres—representing an average of 3 kilos per capita per diem, nearly. It is regrettable that our profession should manifest race prejudice, even in time of war, to such a degree and in such a manner. Much of the article, however, consists of specific charges of unsanitary, filthy and deliberately indecent abuse of residences, stores and factories, and even churches occupied by German troops. In some instances, these practices are charged to officers and indicate phases of sadism. It is almost inconceivable that such practices are representative of the German army and, we venture to suggest that, instead of considering them as matters of discussion bearing on the physiology and psychology of a race, this society should "carry the war into Africa" by presenting authenticated examples to the medical staff of the German army, with a formal and courteous protest.

---

The Journal of Laboratory and Clinical Medicine begins with the October, 1915, issue. It is published by the C. V. Mosby Co. of St. Louis at $3.00 per annum (monthly). The editorial staff is as follows: Editor-in-chief, Victor C. Vaughan, University of Michigan, Ann Arbor; associate editors—Pharmacology, Dennis E. Jackson, M. D., Washington University, St. Louis; Bacteriology, Hans Zinsser, M. D., Columbia University, New York; Immunology and Serology, Frederick P. Gay, M. D., University of California, San Francisco; Physiological Pathology, Paul G. Woolley, M. D., University of Cincinnati, Cincinnati; Physiological Chemistry and Clinical Physiology, J. J. R. MacLeod, M. D., Western Reserve University, Cleveland; Roy G. Pearce, M. D., University of Illinois,, Chicago; Clinical Microscopy and Laboratory Technique, Roger S. Morris, M. D., University of Cincinnati, Cincinnati.

